# Effectiveness of an online education intervention on stress and coping of family members after placing a relative with dementia into a residential care facility: protocol of a randomised controlled trial

**DOI:** 10.1186/s12877-020-01711-8

**Published:** 2020-08-26

**Authors:** Zhaozhao Hui, Chen Yang, Jieqiong Li, Diana Tze Fan Lee

**Affiliations:** 1 The Nethersole School of Nursing, Faculty of Medicine, The Chinese University of Hong Kong, Room 601, 6/F, Esther Lee Building, Hong Kong, SAR China; 2 Department of Nursing, The First Affiliated Hospital of Xi’an Jiaotong University, Xi’an, Shaanxi China

**Keywords:** Dementia, Family members, Stress, Coping, Residential care

## Abstract

**Background:**

After residential care placement, family members may be exposed to stressors like difficulty in role changes, interpersonal conflict with facility staff, and emotional torment. These can threaten family members’ own health and well-being and even influence the extent they involve in their relative’s care. This study aims to evaluate an online education intervention for Chinese family members whose relatives with dementia have been placed into a residential care facility.

**Methods:**

This protocol describes a two-arm randomised controlled trial. A total of 150 family members of residents with dementia will be recruited from four to six residential care facilities in Xi’an, Shaanxi, China and randomly allocated to either the intervention or control group. Family members in the intervention group will receive a six-week group-based online education intervention, while those in the control group will receive routine care. Family members’ stress, coping, caregiving burden, and family involvement, as well as their relative’s behavioural and psychological symptoms of dementia will be assessed at immediately post-intervention and six-week follow-up. Effectiveness of the intervention will be analysed by generalised estimating equation model, based on the intention-to-treat principle. A process evaluation of the intervention will also be undertaken.

**Discussion:**

This study will be of great significance in addressing family members’ stressors after institutionalising a relative with dementia and promoting the implementation of family-centred care in practice especially in residential care facilities.

**Trial registration:**

Chinese Clinical Trial Registry, ChiCTR1900024582, Registered 18 July 2019.

## Background

Previous studies have found that when family caregiving cannot satisfy the care needs of a relative with dementia, family members may choose to place him/her into a residential care facility [1–3]. Alzheimer’s Association has recently revealed that 42% of residents in residential care facilities and 48% of nursing home residents have Alzheimer’s or other dementias [4]. Residential care transition in fact is a stressful experience not only for persons with dementia but also for their family members. For persons with dementia, admission to a residential care facility indicates loss of their familiar surroundings and people [5], and thus may result in a worsening of behavioural and psychological symptoms of dementia (BPSD) [6]. For family members, residential care placement can to some extent reduce their direct care obligations [7, 8]; yet, it does not represent end of the caregiving process and family members’ stress will never be completely eliminated [9, 10]. Guilt, feeling of loss, and feeling of failure have been reported in family members after residential care placement [6, 9–13]. Schulz et al. [12] even found that family members who have institutionalised a relative with dementia experienced as a high level of depressive symptoms and anxiety as that while they provided in-home caregiving.

The philosophy of family-centred care emphases that families are essential allies for quality and safety of health care [14], and health care professionals should dedicate efforts to maintain family members’ control when providing care services for an individual [15]. Over the past two decades, several interventional studies have been conducted to assist family members to cope with stressful situations after placing a relative with dementia into a residential care facility; however, the duration and focus of the interventions varied with each other [16–21]. The *Family Visit Education Programme* (FVEP) in 1999 can be regarded as a pioneer study in this field [16]. This eight-week group-based education intervention was designed to teach primary family visitors on how to effectively communicate and interact with their relatives with dementia during visiting [16]. McCallion et al. [16] found that the FVEP could significantly reduce primary family visitors’ stress in caregiving and residents’ behavioural symptoms, depression, and irritability. Robison and colleagues [17] have also conducted an education intervention, *Partners in Caregiving* (PIC), for family members of nursing home residents with dementia, but focused on communication and cooperation between family members and facility staff. Results showed that after intervention residents’ behavioural symptoms were significantly reduced in the intervention group when compared with controls. But family members’ perceived hassles with staff and caregiving burden in the intervention group were not significantly different from that of the control group [17]. More recently, Paun et al. [18] designed another education intervention, *Chronic Grief Management Intervention* (CGMI) to deliver family members knowledge of dementia, skills in communication, conflict resolution, and hands-on care in the context of long-term care, and grief management skills. This 12-week group-based intervention also showed beneficial effects, with family members who received the intervention reporting significantly lower lever of grief and guilt than those in the control group [18].

Additionally, one psychoeducation intervention [19] and two psychosocial support interventions were also identified [20, 21]. Underpinned by a perspective of empowerment and the model of stress and coping, the 10-week *Taking Care of Myself* psychoeducation intervention employed group sessions to help daughter caregivers feel at ease with their relatives, express the point of views to health care staff, avoid emotional torment, deal with loss, call upon support network, and take care of themselves [19]. Ducharme et al. [19] found that this intervention was effective in reducing daughter caregivers’ stress appraisal and improving their use of coping strategies. With regard to the two psychosocial support interventions, the *Family Intervention: Telephone Tracking-Nursing Home* (FITT-NH) was a purely telephone-delivered intervention based on the stress and coping model. This three-month intervention aimed to identify and address the acute stressors (e.g. family-staff interaction, satisfaction with the facility, and guilt about the placement decision) of family members who were within two months of placing a relative with dementia into a nursing home through 10 telephone contacts [20]. In contrast, the *Residential Care Transition Module* (RCTM) was a multicomponent intervention including psychoeducation, promotion of communication, problem solving, patient behaviour management strategies, concrete goal planning, knowledge about treatments in the residential care facility, as well as ad hoc counselling [21]. Findings indicated that family members receiving the FITT-NH could significantly reduce their feelings of guilt and hassles with facility staff [20], and family members receiving the RCTM intervention reported significantly reduced levels of role overload and caregiving distress [21].

Although current evidence in this field is limited, it can be seen that providing family members with emotion management skills [18–20], enhancing family members’ competence in communicating and interacting with residents [16, 18, 19, 21], teaching family members skills in building positive relationship with facility staff [19, 21], and/or teaching family members strategies to recognise and cope with new roles and ongoing problems after residential care placement [20, 21] can improve family members’ emotional distress (i.e. guilt, role overload, grief) [18, 20, 21], stress appraisal [19], and stress in caregiving [16, 21]. Additionally, improving family-resident communication and interaction or family-staff partnership is promising for reducing residents’ depression [16] and behavioural symptoms [16, 17]. China has the largest number of population with dementia worldwide, and more than half of nursing home residents are reported to be living with dementia [22]. However, no intervention studies to date are available for Chinese family members of people with dementia in residential care facilities. In order to design an intervention for this population, we have conducted a qualitative study in 17 Chinese family members whose relatives with dementia had been placed into residential care facilities to explore their stressors. Findings showed that Chinese family members experienced multiple stressors after placement. Firstly, Chinese family members were criticised as unfilial by people around and thus experienced a high level of guilt and stress after placement. This is mainly because that in the traditional Chinese culture of filial piety older people are supposed to be cared for by the younger generation at home rather than being placed into a residential care facility [23]. Moreover, the facility fees in China were generally high, which to some extent caused a financial burden for family members. Additionally, continuing caregiving at the residential care facilities has aroused a series of difficulties (e.g. communicating with relative with dementia, addressing behavioural problems, building harmonious relationship with facility staff) that created challenges and frustration for family members. Lastly, suboptimal care provided by the facilities also caused worries for family members. As suggested, people in Asian culture tend to adjust to stress with the aim of creating harmony between self and the environment, instead of removing the stressors or changing features of the environment in order to align with one’s needs [24]. In our qualitative study, we also found that Chinese family members were more likely to accept the reality rather than seeking information to resolve problems when faced with stressors.

With the government’s policy inducement, the number of residential care facilities in China has rapidly increased in recent years but the care quality in residential care facilities indeed are still in the developmental stages [25]. Most caregivers working in residential care facilities have insufficient knowledge and skills about dementia care and thus the care they provide is suboptimal [26, 27]. The expenses of living in a residential care facility are not covered by medical insurance in China [28]. Family members’ stress relating to suboptimal care in the facility and high facility fees may need to be resolved from the government level, such as providing standardized dementia care training for care providers and establishing a robust long-term care insurance system [28, 29]. For other identified stressors, we have designed an online education intervention to facilitate Chinese family members reappraise their situations and to enhance their skills in coping with stressful situations after placement. This study aims to evaluate the effectiveness of the online education intervention. The objectives are: (a) to evaluate whether the intervention can reduce stress and improve coping in Chinese family members compared to routine care; (b) to evaluate whether the intervention can reduce caregiving burden and increase care involvement in Chinese family members compared to routine care; and (c) to evaluate whether the intervention can indirectly reduce residents’ BPSD compared to routine care. We hypothesise that at post-intervention and six-week follow-up, family members in the intervention group will report lower level of stress, higher level of use of coping strategies, lower level of caregiving burden, higher level of involvement in their relative’s care, and residents in the intervention group will have lower level of BPSD, when compared to those in the control group.

### Theoretical underpinnings

This study will be underpinned by the model of stress and coping [30–33]. In this model, stress is a particular relationship between the person and the environment that is appraised by the person as taxing or exceeding his or her resources and endangering his or her well-being [31]. When faced with an event, the person firstly evaluates whether it is irrelevant, benign-positive, or stressful for his or her well-being (primary appraisal) [31]. Once the event is judged as stressful (harm/loss, threat, or challenge), a further form of appraisal becomes salient, that of evaluating what can be done to overcome or prevent harm or to improve prospects for benefit (secondary appraisal) [30, 31]. In appraisal, event uncertainty has a great potential for creating psychological stress [31]. It can be somewhat interpreted as that the more uncertain the person perceives about an event, the more likely he or she appraises it as stressful. Moreover, the extent to which a person believes he or she can handle the person-environment relationship (beliefs about personal control) affects whether and to what extent the person feels stressful in that encounter [31].

Faced with a stressful situation, the person will make efforts to regulate stressful emotions to the problem (emotion-focused coping) and/or manage or alter the troubled person-environment relation causing the stress (problem-focused coping) [33]. In the process of coping, the person’s problem-solving skills and social support can serve as important coping resources [31]. Coping is constantly changing cognitive and behavioural efforts to manage specific demands that are appraised as taxing or exceeding the recourses of the person [31]. If coping results are unfavourable, the person then may change to meaning-focused coping (e.g. benefit finding). Meaning-focused coping is, in its essence, appraisal-based coping in which the person draws on his or her beliefs and existential goals to motivate and sustain coping and well-being during a difficult time [32].

Based on the model of stress and coping, the development of efficacious interventions for reducing a person’s stress should dedicate efforts to cognitive appraisal of the person-environment relationship and available coping resources and use of coping strategies. Interventions to facilitate family members to reappraise the situations (reappraisal/meaning-focused coping), to manage negative emotions (emotion-focused coping), and to deal with stressful situations (problem-focused coping) after residential care placement are anticipated to result in positive outcomes.

## Methods

### Study design

**Fig. 1:**
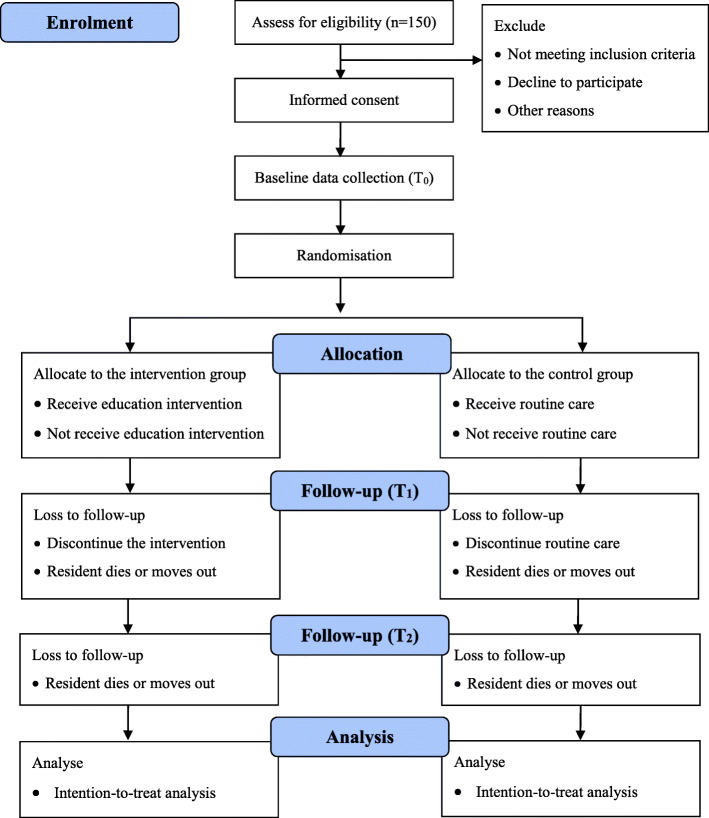
CONSORT flow diagram of the trial

The protocol describes a multicentre, two-arm, assessor-blind randomised controlled trial (RCT), with randomisation of family members of residents with dementia rather than residential care facilities. The CONSORT flow diagram is shown in Fig. 1. Although there might be crossover effects between family members within a residential care facility in which some belong to the intervention group and others to the control group, we still choose an individual RCT design because of its ability to deal with known and unknown confounding factors. To avoid the contamination between participants of the two groups, some strategies will be employed, such as asking participants in the intervention group not to share the intervention information with others.

### Participants and recruitment

Family members of residents with dementia will be recruited from four to six medium-sized residential care facilities (100–300 beds) in Xi’an, Shaanxi, China. Posters will be available in the studied facilities to encourage more interested and eligible family members to participate in the trial. Potentially eligible and interested participants will be referred to the researchers by nurses who work in the studied facilities. The researchers will then approach the potentially eligible participants to determine their eligibility and invite them to participate in this trial. Information sheets will be provided to the eligible participants and any questions or inquiries will be answered by the researchers.

To be eligible, the participants should (a) be family members of residential care facility resident who has a medical diagnosis of dementia, (b) be aged 18 years or older, (c) provide the most hours to their relative’s care after placement, (d) have a smartphone and be able to use it, (e) be willing to participate in this study, and (f) be able to read, write and understand Chinese. Exclusion criteria of the participants are: (a) having severe mental disorders (e.g. schizophrenia), (b) having impaired cognitive ability, (c) being a family member whose relative with dementia is at end of life (i.e. the life expectancy is projected to be six months or less), (d) being a paid caregiver, (e) receiving psychotherapies during the period of this study, and (f) participating in other relevant psychosocial interventions or programmes during the period of this study. Participant recruitment for this trial is currently ongoing.

### Sample size estimation

Sample size is estimated based on the primary outcomes, specifically, changes in family members’ stress. In this study, effect size of family members’ stress reduction is anticipated to be medium at 0.50. It is estimated that a sample size of 128 participants (64 per group) will provide this study with 80% power at 5% level of significance (two-tailed). Attrition rates in previous studies ranged from 0% [21] to 34.3% [34], most of which were less than or around 15% [16–18, 20]. In consideration of 15% attrition rate, sample size of this study is finally estimated to be 150 family members (75 per group).

### Randomisation and allocation concealment

Randomisation will be carried out using computer-generated random numbers (http://www.randomization.com). One research assistant will be responsible for generating the random numbers and putting them into sequentially numbered opaque sealed envelopes. This research assistant will not involve in participant recruitment, intervention implementation, outcome assessment, and/or data analysis. The eligible participants will be randomly assigned to either the intervention group (odd numbers) or the control group (even numbers) according to the random numbers.

### Blinding

Participants and implementer(s) of the intervention will not be blinded to the participants’ group allocation. Outcome assessors will be blinded to the participants’ group allocation until the entire data analysis has been completed.

### Intervention group

Participants in the intervention group will receive a six-session group-based (6 to 10 participants) online education intervention. The intervention will be delivered on a weekly basis, approximately 60 min per week. Participatory approaches (e.g. experience sharing, brainstorm) will be used during the intervention delivery. Participants will be encouraged to complete the whole intervention through text message reminders; yet the situation that participants discontinue the allocated intervention because their relative with dementia dies or moves out of the studied facilities will be allowed.

Session 1 aims to facilitate the participants to reappraise the situations they are facing with, through improving their understanding of dementia. Specifically, the researcher will provide the participants with basic dementia knowledge especially its progression and manifestations at different stages. These kinds of information are anticipated to reduce the participants’ uncertainty about dementia, which may further facilitate the participants to reappraise the situations they are facing with.

Session 2 aims to help the participants manage negative emotions after residential care placement. Firstly, the participants will be encouraged to share their experiences of managing negative emotions after residential care placement. The researcher will introduce the participants some emotion management strategies (e.g. accepting the reality, benefit finding, and humour). In particular, the researcher will guide the participants to find benefits of residential care placement in order to help them reframe stressful situations by wresting positive values form adversity.

Sessions 3 to 5 aim to improve the participants’ problem-solving skills in controlling or managing the stressful situations during visits. In session 3, the researcher will impart the participants communication tips with people with dementia (e.g. approaching from the front, minimising distraction, asking short and answerable questions). In session 4, the researcher will guide the participants to analyse the causes or underlying meanings of BPSD in people with dementia. Strategies of how to deal with BPSD (e.g. delusion, hallucination, agitated behaviour, wandering, irritability, anxiety, appetite changes, and inappropriate sexual behaviour) during visits will be introduced to the participants. In session 5, the researchers will help the participants to recognise their role changes and potential benefits of family involvement after residential care placement. Key points of establishing harmonious family-staff partnership (e.g. information sharing, setting realistic expectations) will be also introduced to the participants.

Session 6 aims to help the participants identify and call upon social support, which is an important coping resource when encountering stressful situations. The participants will be invited to depict their available social support network and will be encouraged to call upon it when encountering stressful situations. Additionally, approaches to resolving conflicts with other family members (e.g. organising a family conference) will be provided to the participants.

### Control group

Family members allocated to the control group will be allowed to use community-based resources and irregularly chat with the facility staff but will not be provided with additional interventions, therefore reflecting the current routine care.

### Outcome measures

**Fig. 2:**
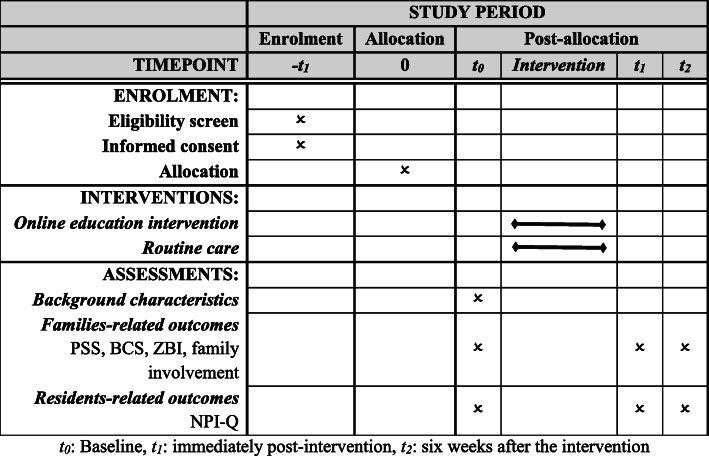
Enrolment, interventions, and assessments of the trial (PSS: Perceived Stress Scale, BCS: Brief COPE Scale, ZBI: Zarit Burden Interview, NPI-Q: Neuropsychiatric Inventory-Questionnaire). *t*_*0*_: Baseline, *t*_*1*_: immediately post-intervention, *t*_*2*_: six weeks after the intervention

Outcome measures will be collected at baseline (t_0_), immediately post-intervention (t_1_), and six weeks after intervention (t_2_), by two trained research assistants (Master students of Nursing). To collect six-week follow-up outcome data, the researchers will reminder all the participants via text messages. The schedule of enrolment, interventions, and assessments of the trial is shown in Fig. 2, as recommended in the SPIRIT 2013 statement [35].

### Background characteristics

Sociodemographic characteristics of family members and their relative with dementia will be collected at baseline using a self-developed questionnaire. Data for family members include age (years), gender, ethnicity, educational attainment (primary school or lower, middle school, high school, or college/university), marital status (unmarried, married, divorced, or others), relationship with the resident (spouse, child, child-in-law, grandchild, or others), employment status (full-time, part-time, retired, or unemployed), whether lived with the resident before placement, and the proportion of facility fees that the family member or his/her family need to pay (almost zero, less than 20, 20 to 50%, 51 to 80%, or more than 80%). Data for residents with dementia include age (years), gender, ethnicity, educational attainment (primary school or lower, middle school, high school, or college/university), marital status (unmarried, married, divorced, or others), and length of stay in the studied facility (months).

In addition, for residents with dementia, disease-related data such as type of dementia (Alzheimer’s disease, vascular dementia, Lewy body disease, Parkinson’s disease, frontotemporal dementia, or others), time of diagnosis, and cognitive ability (e.g. Mini-Mental State Examination), will be extracted from the medical records.

### Stress

Family members’ stress will be measured using the 10-item Perceived Stress Scale (PSS-10), which was designed to tap how unpredictable, uncontrollable, and overloaded respondents find their lives during the last month [36, 37]. The questions in the PSS-10 adopt a five-point Likert scale ranging from 0 (never) to 4 (very often). The total score of the PSS-10 ranges from 0 to 40; a higher score suggests a higher level of stress. The Chinese version of PSS-10 has been validated, indicating a satisfactory internal consistency reliability for evaluating stress levels in Chinese population (Cronbach’s α = 0.86) [38].

### Coping strategies

Family members’ use of coping strategies will be measured using the 28-item Brief COPE Scale (BCS) [39]. This scale consists of 14 domains and adopts a four-point Likert scale ranging from 1 (I have not been doing this at all) to 4 (I have been doing this a lot). The total score of the BCS scale ranges from 0 to 112; higher scores indicate more frequent use of coping strategies. The Chinese version of the BCS has been validated, suggesting a satisfactory internal consistency reliability for evaluating use of coping strategies in Chinese population (Cronbach’s α = 0.79) [40].

### Caregiving burden

Family members’ caregiving burden will be measured using the 22-item Zarit Burden Interview (ZBI) [41]. This instrument covers family members’ health, psychological well-being, social life, finances, and relationship between the family members and their relative [41]. Each item of the ZBI is answered on a five-point scale (0 = never, 1 = rarely, 2 = sometimes, 3 = quite frequently, and 4 = always). The total score of the ZBI ranges from 0 to 88, with a higher score indicating a higher level of burden. The Chinese version of the ZBI shows a sound internal consistency reliability in Chinese family caregivers (Cronbach’s α = 0.875) [42].

### Family involvement

Family involvement will be assessed by several simple questions referring to Roberts, Ishler, & Adams [43]. The questions cover family visiting, providing personal care, and communicating with facility staff [44]. For visiting, families will be asked to estimate their average frequency of visiting the nursing home using these response options: daily, several times per week, once per week, two or three times per month, once per month, or a few times per year. They will be also asked how often they have helped their relative with dementia during visits in feeding, dressing, toileting, grooming, and going to activities on a scale of “never” (0), “sometimes” (1), or “always” (2). Responses to these five activities will be summed to indicate family involvement in providing personal care. For communication with facility staff, family members will be asked how often they have talked with four categories of facility staff members during the last month: care workers, nurses, physicians, and administrators on a scale of “never” (0), “sometimes” (1), and “always” (2). A measure of the overall level of communication with facility staff will be created as a sum of these responses. These questions have been translated into Chinese by the researchers for this study.

### BPSD

Residents’ severity of BPSD will be assessed by inquiring their primary nurse using the 12-item Neuropsychiatric Inventory-Questionnaire (NPI-Q) [45], which can also measure the impacts of BPSD to caregivers. Initial responses to each question are “yes” or “no”. If “yes”, the respondents will then be asked to rate the frequency and severity of the symptoms as well as the associated impacts. A higher score indicates a more severe BPSD and a higher level of caregiver distress. The Chinese version of the NPI-Q has been validated, suggesting a satisfactory reliability in Chinese residential care facility residents with dementia (Cronbach’s α = 0.64, intra-class correlation coefficient = 0.93) [46].

### Intervention fidelity and process evaluation

The fidelity of intervention implementation will be monitored and assessed. Prior to conducting the trial, an exhaustive protocol will be developed to guide implementation of the intervention. A checklist will be also developed for evaluating the intervention fidelity. The duration of each session and the attendance of the participants in each session will be recorded. Furthermore, outcome assessors will be trained to deliver the standardised assessment measures.

### Data management and analysis

Data will be double entered and checked in EpiData 3.1 (The EpiData Association, Odense, Denmark) and will then be exported to Statistical Package for the Social Sciences (SPSS) version 25.0 (IBM Corporation, Armonk, New York, USA) for analyses. The distribution normality of the continuous variables will be tested by One-Sample Kolmogorov-Smirnov test. The baseline homogeneity between the intervention group and the control group will be tested using the Student’s T test, Mann-Whitney U test, Chi-square test, and Fisher’s exact test, where appropriate. All outcome measures will be compared within and between groups. The generalised estimating equation (GEE) model will be utilised to analyse the intervention effects, with controlling for confounding factors such as the family members’ age, gender, educational attainment, and relationship with the residents, and the residents’ age, gender, educational attainment, type of dementia, and length of stay in the facility. The analyses will be performed based on the intention-to-treat (ITT) principle. For ITT analysis, missing data will be dealt by using the last observation carried forward method, whereby the last available measurement for each participant at the time point prior to withdrawal from the study is retained in the analysis [47]. Statistical significance will be set at *p* < 0.05; all tests will be two-tailed.

## Discussion

Family members usually face challenges and stressors after institutionalising a relative with dementia, this study will evaluate an online education intervention for Chinese family members of residential care facility residents with dementia. This will have implications for nursing research and practice. Firstly, with a mixed methods experimental study design, an online education intervention has been developed based on the Chinese family members’ own perceptions and expectations after placing a relative with dementia into a residential care facility. This will provide evidence to health care professionals and researchers in designing and implementing more successful interventions for this group of population. Secondly, interventions to family members that aim to address their stressors may influence the extent they involve in their relative’s care and thus indirectly affect resident outcomes. This study will shed insight on the effectiveness of an education intervention in terms of both family members and residents with dementia. Thirdly, this study will be of great significance in promoting the implementation of family-centred care in practice especially in residential care, and improving family involvement in their relative’s care after residential care placement.

The results of this study will be presented at national and/or international conferences. Findings pertaining to this study will be submitted and published in peer reviewed scientific journals. If the results of this study demonstrated beneficial effects, the intervention will be disseminated to more local residential care facilities.

## Data Availability

The datasets and any materials used and/or analysed during the current study will be available from the corresponding author on reasonable request.

## Abbreviations

BPSD: Behavioural and Psychological Symptoms of Dementia
FVEP: Family Visit Education Programme
PIC: Partners in Caregiving
CGMI: Chronic Grief Management Intervention
FITT-NH: Family Intervention: Telephone Tracking-Nursing Home
RCTM: Residential Care Transition Module
RCT: Randomised Controlled Trial
PSS: Perceived Stress Scale
BCS: Brief COPE Scale
ZBI: Zarit Burden Interview
NPI-Q: Neuropsychiatric Inventory-Questionnaire
SPSS: Statistical Package for the Social Sciences
GEE: Generalised Estimating Equation
ITT: Intention-to-treat
